# Implementation Science Research Examining the Integration of Evidence-Based Practices Into HIV Prevention and Clinical Care: Protocol for a Mixed-Methods Study Using the Exploration, Preparation, Implementation, and Sustainment (EPIS) Model

**DOI:** 10.2196/11202

**Published:** 2019-05-23

**Authors:** April Idalski Carcone, Karin Coyle, Sitaji Gurung, Demetria Cain, Rafael E Dilones, Laura Jadwin-Cakmak, Jeffrey T Parsons, Sylvie Naar

**Affiliations:** 1 Department of Family Medicine and Public Health Sciences Wayne State University Detroit, MI United States; 2 Education, Training, and Research Scotts Valley, CA United States; 3 Center for HIV Educational Studies and Training Hunter College City University of New York New York, NY United States; 4 Department of Health Behavior and Health Education University of Michigan School of Public Health Ann Arbor, MI United States; 5 Hunter Department of Psychology Hunter College City University of New York New York, NY United States; 6 Health Psychology and Clinical Science Doctoral Program Graduate Center City University of New York New York, NY United States; 7 College of Medicine Florida State University Tallahassee, FL United States

**Keywords:** implementation science, HIV, evidence-based practice, motivational interviewing

## Abstract

**Background:**

The Exploration, Preparation, Implementation, and Sustainment (EPIS) model is an implementation framework for studying the integration of evidence-based practices (EBPs) into real-world settings. The EPIS model conceptualizes implementation as a process starting with the earliest stages of problem recognition (Exploration) through the continued use of an EBP in a given clinical context (Sustainment). This is the first implementation science (IS) study of the integration of EBPs into adolescent HIV prevention and care settings.

**Objective:**

This protocol (ATN 153 EPIS) is part of the Scale It Up program, a research program administered by the Adolescent Medicine Trials Network for HIV/AIDS Interventions (ATN), described in this issue by Naar et al. The EPIS study is a descriptive study of the uptake of 4 EBPs within the Scale It Up program. The goal of EPIS is to understand the barriers and facilitators associated with the Preparation, Implementation, and Sustainment of EBPs into HIV prevention and clinical care settings.

**Methods:**

The EPIS study is a convergent parallel mixed-methods IS study. Key implementation stakeholders, that is, clinical care providers and leaders, located within 13 ATN sites across the United States will complete a qualitative interview conducted by telephone and Web-based surveys at 3 key implementation stages. The Preparation assessment occurs before EBP implementation, Implementation occurs immediately after sites finish implementation activities and prepare for sustainment, and Sustainment occurs 1 year postimplementation. Assessments will examine stakeholders’ perceptions of the barriers and facilitators to EBP implementation within their clinical site as outlined by the EPIS framework.

**Results:**

The EPIS baseline period began in June 2017 and concluded in May 2018; analysis of the baseline data is underway. To date, 153 stakeholders have completed qualitative interviews, and 91.5% (140/153) completed the quantitative survey.

**Conclusions:**

The knowledge gained from the EPIS study will strengthen the implementation and sustainment of EBPs in adolescent prevention and clinical care contexts by offering insights into the barriers and facilitators of successful EBP implementation and sustainment in real-world clinical contexts.

**International Registered Report Identifier (IRRID):**

DERR1-10.2196/11202

## Introduction

### Background

Over the past 25 years, behavioral scientists have developed a number of efficacious interventions to reduce HIV transmission and improve self-management among those living with HIV. Between 2003 and 2014, the overall incidence of HIV in the United States decreased by 25%, yet youth aged 13 to 24 experienced a 43% increase [1] and accounted for a quarter (26%) of new HIV infections. More than half of (60%) of youth living with HIV are unaware of their HIV status. Once diagnosed, less than two-thirds are linked to HIV clinical care within 1 year, and just over half (54%) achieve viral suppression. Hence, fewer than 10% of US youth are and remain virally suppressed [2]. These data clearly illustrate that implementation of efficacious interventions in settings that serve youth has not yet been fully realized.

Implementation science is the study of methods and factors influencing the translation of research and other evidence-based practices (EBPs) into routine care [3]. Multiple implementation theories and models have been proposed for the prediction or explanation of the process of adopting and sustaining EBPs within the social sector. Where theories seek to generalize predictable pathways of translating knowledge into practice, determinant models and frameworks attempt to explain the factors that influence various stages of adoption, implementation, and sustainability in specific fields and contexts [4]. Determinant models originating from child welfare and mental health fields may be particularly pertinent to the HIV field because of the similar ways in which social context influences program delivery to youth and the adoption of new practices by the clinical care providers.

The Exploration, Preparation, Implementation, Sustainment (EPIS) model [5,6] is an implementation framework studying the integration of EBPs into real-world settings. A strength of the EPIS model is its view of EBP implementation across 4 phases [7]. The Exploration phase involves the recognition of a concern or opportunity for improvement. In Preparation, there is a decision to adopt an EBP. Implementation refers to the active integration of the EBP into routine care, whereas Sustainment examines the continued use of the new EBP. Within each phase, EPIS outlines and highlights the interplay between critical inner (internal to the organization, eg, organizational leadership and clinician characteristics) and outer (external systems, eg, political environment, funding, and other resources) contextual factors likely to impact EBP implementation. A number of reliable, validated measures of these inner and outer contextual factors have been published in the research literature (see Measures section for a description of selected measures), making the EPIS model an ideal framework for the study of EBP implementation in HIV clinical care settings [8,9]. Finally, the EPIS model has been successfully used to study EBP uptake in similar multisite effectiveness trials such as the JJ-TRIALS and SAT2HIV [10-12] studies.

### Aims and Objectives

This paper describes the EPIS research protocol, a study being conducted by the Adolescent Medicine Trials Network for HIV/AIDS Interventions (ATN; referenced as ATN 153 EPIS). EPIS is a mixed-methods implementation science (IS) research study of the uptake of 4 EBPs across the United States at ATN research sites. Thus, EPIS is 1 study within a larger program of research, “Scale It Up,” to improve HIV-related self-management among youth living with or at risk of contracting HIV [13]. The 4 EBPs include sequential multiple assignment randomized trial (referred to as ATN 144 SMART), an adaptive intervention that combines short message service text messaging and cell phone support to increase antiretroviral therapy adherence among youth living with HIV (see the study by Belzer et al [14] in this issue). Scale It Up also includes a comparative effectiveness trial of clinic- versus telephone-delivery of the Young Men’s Health Project (referred to as ATN 145 YMHP), a 4-session intervention to reduce the risk of HIV infection among young men who have sex with men (see the study by Parsons et al [15] in this issue). The tailored motivational interviewing (MI) study (referred to as ATN 146 TMI) aims to scale up the use of an EBP, MI, in adolescent HIV clinical care settings (see the study by Naar et al [16] in this issue). Finally, a comparative effectiveness trial to assess the additive benefit of communication training during couples’ HIV testing and counseling (referred to as ATN 156 We Test; see the study by Starks et al [17] in this issue). The goal of the EPIS study is to describe the inner and outer contextual factors impacting the uptake of these 4 EBPs across 3 implementation phases. In years 1 to 2, as sites prepare for the integration of EBPs into their clinical care routines, the EPIS study will assess several providers and organizational characteristics that may impact the implementation and sustainment of EBPs at each clinical site (Table 1). Years 3 to 4 will focus on understanding the barriers and facilitators sites experienced during Implementation, and year 5 will assess plans for Sustainment.

**Table 1:** Exploration, Preparation, Implementation, and Sustainment (EPIS) model Inner (I) and Outer (O) context factors to be explored in the EPIS protocol.

Factors	Data source	EPIS phase/timeline for data collection		
		Preparation (Years 1 to 2)	Implementation (Years 2 to 3)	Sustainment (Years 4 to 5)
Leadership (I^a^)	Survey	✓^b^	✓	✓
Organizational culture and climate (I)	Interviews; survey	✓	✓	✓
Fiscal viability and resources (I, O^c^)	Interviews; survey	✓	✓	✓
Experience with evidence-based practices (I)	Interviews	✓	✓	✓
Attitudes toward evidence-based practices, including perceived barriers and facilitators (I)	Interviews; survey	✓	✓	✓
Facilitator/provider characteristics (I)	Survey	✓	✓	✓
Intervention fit (I)	Interviews; survey	✓	✓	✓
Interorganizational networks (O)	Interviews	✓	✓	✓
Fidelity monitoring and support^d^	Clinical records	—^e^	✓	✓
Perceived client outcomes	Interviews	✓	✓	✓

^a^I: inner context factor.

^b^Factor collected at a given EPIS phase/timeline.

^c^O: outer context factor.

^d^Fidelity data (defined as the extent to which providers adhere to treatment protocols) will be collected as part of the Scale It Up individual study protocols.

^e^Not applicable.

## Methods

### Design

This study will use a convergent parallel mixed-methods design [18] with data collected at 3 critical implementation phases: preimplementation (Prepare), postimplementation (Implementation), and sustainment. Participants will be enrolled in the EPIS study for up to 40 months. Preimplementation interviews will be conducted before EBP implementation, beginning in June 2017 and concluding in March 2018. The postimplementation interviews are scheduled to coincide with the sites’ completion of the implementation phase, beginning in March 2019. Sustainment interviews will begin in March 2020 to capture participant perceptions of sustainment 1 year postimplementation. At each phase, participants will complete a qualitative interview by telephone and a quantitative survey via electronic data capture. Questions will focus on participants’ perceptions of the barriers and facilitators to EBP implementation within their clinical site as outlined by the EPIS model.

### Participants and Targeted Sites

All medical providers and staff with patient contact (“Key Stakeholders”) at 13 ATN sites participating in the aforementioned Scale It Up research projects will be eligible to participate (Table 2). Patient contact is defined as having direct patient interaction across several points of care, including prevention, counseling and testing, linkage to care, HIV primary care, services to promote retention and adherence to medications, and other medical or psychosocial services. Key stakeholders will also include administrative and research staff with key decision-making roles (eg, division chief and clinic director) who will provide input on prevention and care services and site operations. Each site will identify a clinical leader (“Site PI”) to represent the organizational leadership perspective. There are no exclusion criteria. Participant turnover will be managed by maintaining the participant’s responses collected up to the point of separation as a part of the study data corpus, but participants will not be retained in the study post separation. Similarly, if a site discontinues its participation, participants associated with that site will remain part of the study data corpus. Newly hired medical providers and staff at the 2 follow-up points will be invited to participate. Different sites participated in different Scale It Up projects because of the differing nature of each EBP being tested and the hybrid design selected for each effectiveness-implementation trial (see the study by Naar et al [13] in this issue). For example, ATN 146 used providers as the participants, but the other 3 trials primarily used patients as the unit of analysis.

**Table 2:** Scale It Up projects and participating sites in the Exploration, Preparation, Implementation, and Sustainment protocol.

Site	City, State	ATN^a^ 144 SMART^b^	ATN 145 YMHP^c^	ATN 146 TMI^d^	ATN 156 We Test
Johns Hopkins University	Baltimore, MD	X^e^	—^f^	X	—
University of Alabama at Birmingham/Birmingham AIDS Outreach	Birmingham, AL	X	—	X	—
Center for HIV Educational Studies and Training at Hunter College^g^	New York, NY	—	—	—	X
State University of New York Downstate Medical Center	Brooklyn, NY	X	—	X	—
Wayne State University Prevention	Detroit, MI	—	X	—	X
Children’s Hospital of Los Angeles	Los Angeles, CA	X	—	X	—
St. Jude Children’s Research Hospital	Memphis, TN	X	—	X	—
University of Miami	Miami, FL	X	X	X	X
Tulane University^h^	New Orleans, LA	X	—	X	—
Children’s Hospital of Philadelphia	Philadelphia, PA	X	X	X	—
University of California, San Diego	San Diego, CA	X	—	X	X
University of South Florida	Tampa, FL	X	—	X	—
Children’s National Health System	Washington, D.C.	X	—	X	—

^a^ATN: Adolescent Medicine Trials Network for HIV/AIDS Interventions.

^b^SMART: Sequential Multiple Assignment Randomized Trial.

^c^YMHP: Young Men’s Health Project.

^d^TMI: Tailored Motivational Interviewing Implementation Intervention.

^e^Site is participating in given SIU project and receives relevant questions for Exploration, Preparation, Implementation, and Sustainment model.

^f^Not applicable.

^g^Postimplementation and sustainment phase only.

^h^Preimplementation phase only.

Before each data collection effort, each site will provide a list of the medical providers and staff with direct patient contact. This list will include names, contact information (phone number and email), and role(s) within the clinic. Potential participants will receive an initial “enrollment email” introducing them to the EPIS model and study and providing them with instructions for scheduling their qualitative interview through a Web-based scheduling system. After the initial email, potential participants will be sent reminders every 2 weeks throughout the baseline study period about project enrollment. All sites have agreed to permit participants to participate in EPIS data collection efforts during their regularly scheduled work hours. Participants will be provided a list of available interview times from which they can choose an interview time most convenient for their schedule and availability. Participants will also be given the option of directly emailing their availability to arrange the most convenient interview. Interviewers are centralized, providing available times for all sites and will call participants at the scheduled time.

Upon completion of the interview, participants receive a link to complete the survey in Qualtrics. Participants who complete both the qualitative interview and quantitative survey receive a US $10 Amazon e-gift card. If a participant completes all 3 assessments (ie, preimplementation, implementation, and sustainment), they can receive a total of US $30 in Amazon e-gift cards. Participants who have not completed the quantitative survey will receive periodic reminders to do so for the duration of the data collection window.

All study procedures were approved by the institutional review board of the Scale It Up principal investigator’s (PI) academic institution. All participants provided oral informed consent before the initiation of any study activity.

### Assessments

Assessments will elicit participants’ perceptions of barriers and facilitators to EBP implementation and sustainment at 3 critical implementation phases: preimplementation (Prepare), postimplementation (Implementation), and sustainment. The baseline assessment (June 2017-March 2018) will capture preimplementation feedback on anticipated barriers and facilitators for the specific EBPs each site will be implementing. The first follow-up assessment will occur postimplementation (March 2019-February 2020) and will assess barriers and facilitators experienced during EBP implementation and query anticipated barriers and facilitators to sustaining the EBPs. The second follow-up assessment (March 2020-February 2021) will assess barriers and facilitators experienced during the initial (1 year postimplementation) sustainment period.

#### Interviews

Trained interviewers will conduct interviews by telephone using a semistructured interview guide. Interview domains will include gathering information about the participant’s professional background and experience, clinical site organization and structure, familiarity with EBPs in general, familiarity with the specific EBPs being implemented, and perceived barriers and facilitators to implementing the specific EBPs. In addition, site PIs will be asked about organizational history with EBPs, internal (organizational) and external (community and state) leadership structures, and their site’s political context (policies and funding mechanisms) and fiscal considerations. It is estimated that key stakeholder interviews will require 30 min to 60 min to complete. Site PI interviews will require 60 min to 90 min to complete and thus will be completed in 2 parts (30 min to 60 min each).

Interviewer training will include prework for priming before the training and a 2-part live virtual training with modeling. Follow-up support will include interviewers conducting 2 mock interviews with self-assessment and trainer ratings and feedback following each mock interview; the rating forms were adapted from the study by Amico [19]. Interviewers who do not achieve adequate ratings on the second mock interview will complete a third to determine if they are fit for the interviewer role. Once data collection begins, the project team will hold monthly interview support calls that focus on reviewing and problem-solving issues raised by interviewers or identified through a review of transcripts. Interviewers will also be able to trigger immediate support through a Web-based technical assistance support form. Training procedures will be initiated 1 month before each data collection point.

Interviews will be audio-recorded and, immediately upon completion of the interview, uploaded to a secure server for storage. Audio files will be electronically transferred to a professional transcription service. Transcriptionists will provide a verbatim, deidentified transcript of the interview. Deidentification will involve removing participant and clinic staff member names. Research staff will review transcripts for quality (ie, accuracy) and confidentiality (ie, deidentification) before releasing the data for coding. Interview data will be uploaded to NVivo Version 12 (QSR International, Inc) for analysis.

#### Survey

Key stakeholders’ and Site PIs’ attitudes toward the adoption of EBPs will be assessed with the *Evidence-Based Practice Attitude Scale* (EBPAS; Aarons) [20]. The EBPAS assesses 4 attitudinal dimensions with strong internal consistency reliability: intuitive *Appeal* of EBP (alpha=.80), likelihood of adopting EBP given *Requirements* to do so (alpha=.90), *Openness* to new practices (alpha=.78), and perceived *Divergence* from usual practice with research-based / academically developed interventions (alpha=.59). They will also complete an updated version of the scale, the *Evidence-Based Practice Attitude Scale-50* (EBPAS-50; Aarons et al), which assesses 8 additional attitudinal domains [21]. The EBPAS-50 assesses the following: EBPs *Limitations* and their inability to address client needs (alpha=.92), EBP *Fit* with the values and needs of the client and clinician (alpha=.88), negative perceptions of *Monitoring* or supervision (alpha=.87), the *Balance* of skills and the role of science in treatment (alpha=.79), time and administrative *Burden* associated with learning EBPs (alpha=.77), likelihood of increased *Job Security* or professional marketability provided by learning an EBP (alpha=.82), *Organizational Support* for learning an EBP (alpha=.85), and positive perceptions of receiving *Feedback* related to service delivery (alpha=.82).

Participants’ perceptions of organizational climate will be assessed with 3 measures. Key stakeholders’ and Site PIs’ perceptions of organization climate, in general, will be assessed with the *Organizational Climate Measure* (OCM; Patterson et al) [22]. The OCM assesses organizational policies, practices, and procedures that provide a contextual backdrop for interactional patterns and behaviors that foster creativity, innovation, safety, or service within the organization, in other words, teamwork. Subscales will include the emphasis given to *Quality* procedures (alpha=.80), *Training* or a concern with developing employee skills (alpha=.83), and *Performance Feedback* (alpha=.78), which refers to the measurement and feedback of job performance. They will also complete the *Implementation Climate Scale* (ICS; Ehrhart et al) [23]. The ICS reliably assesses the extent to which a clinic fosters EBP implementation across 6 dimensions: *Focus* on EBP (alpha=.91), *Educational Support* for EBP (alpha=.84), *Recognition* for EBP (alpha=.88), *Rewards* for EBP (alpha=.81), *Selection* for EBP (alpha=.89), and *Selection* for openness (alpha=.91). Key stakeholders will only complete the *Perceived Organizational Support Scale* (POS; Rhoades et al) [24]. The POS assesses general beliefs about the extent to which an organization values employees’ contributions and cares about their well-being (alpha=.90).

Key stakeholders and Site PIs will also evaluate the role of leadership in the implementation of EBPs using 2 scales: the *Director Leadership Scale*, (DLS; Broome et al) [25] and the *Implementation Leadership Scale* (ILS; Aarons et al) [26]. The DLS is a brief global assessment of organizational leadership with strong internal consistency (alpha=.90). The ILS assesses strategic leadership support for EBP implementation with 4 subscales: *Proactive* leadership (alpha=.95), *Knowledgeable* leadership (alpha=.96), *Supportive* leadership (alpha=.95), and *Perseverant* leadership (alpha=.96).

The extent to which the strategies, procedures, and elements of the 4 EBPs being implemented in the Scale It Up program match the values, needs, skills, and available resources (contextual fit) will be assessed with an adapted version of the *Self-Assessment of Fit in Schools* [27]. Key stakeholders and Site PIs will rate the extent to which they have the skills required to implement the EBPs, their comfort with the different elements of the EBPs, consistency of the EBPs with current clinical practices, ease of implementation including availability of resources and administrative support for the implementation of the EBPs, and perceived efficacy of the EBPs.

Site PIs will assess the extent to which their staff contributes to EBP implementation by demonstrating behaviors that go beyond minimum requirements using the *Implementation Citizenship Behavior Scale* (ICBS; Ehrhart et al) [28]. The ICBS assesses 2 domains: helping others (alpha=.93) and keeping informed (alpha=.91). Finally, all participants will complete an investigator-developed survey to collect basic demographic information, such as position, years in position, race, ethnicity, gender identity, and current caseload. It is estimated that it will require participants 60 min to 90 min to complete the survey.

### Analysis Plan

The analyses will focus on understanding the barriers and facilitators located within sites’ inner and outer context that is associated with implementing and sustaining EBPs into HIV care settings. Analyses will be guided by the following questions: (1) How do inner context factors (eg, organizational culture and climate and leadership) influence EBP implementation and sustainment? (2) How do outer context factors (eg, fiscal viability and interorganizational networks) influence EBP implementation and sustainment? (3) To what extent do the perceptions of key stakeholders and clinical leaders (ie, site PIs) vary, and how does that variation affect EBP implementation and sustainment? (4) To what extent do stakeholder perceptions (key stakeholder and site PI combined) vary by site (ie, organizational structure)?

#### Qualitative Analysis Plan

First, consistent with Morgan’s [29] recommendations for qualitative content analyses and Hsieh and Shannon’s [30] directed qualitative content analytic approach, standard definitions of the concepts of interest will be developed on the basis of the EPIS model. Each interview will be systematically reviewed at each time point for all thematic mentions of the following: (1) features of the inner and outer context per EPIS that have the potential to influence implementation of an EBP, (2) people who have the potential to influence implementation of an EBP, and (3) personal perceptions of the EBP in question and other EBPs that have the potential to improve patient outcomes. Within these longer thematic lists, we will then separate out specific categories of work-setting characteristics (eg, leadership, incentives, and disincentives for innovating), people (eg, patients, nurses, physicians, administrators, experts, and novices), and perceptions of evidence-based interventions (eg, feasible and advantageous), initially using existing theory to guide categorization but also allowing themes to emerge from the data through open coding procedures [31,32]. This combined inductive and deductive coding approach will allow us to both validate and extend the EPIS model. Revision of our initial coding categories will occur iteratively until we reach saturation in the identification of new codes. During this iterative process, categories and their definitions will be refined and subcategories of codes will be consolidated, consistent with an axial-coding process. At this point, we will return to each interview and systematically apply the final, revised set of codes. In addition, case codes will be applied to each interview to reflect clinic role, site, cluster, and relevant demographic characteristics of the respondent.

The coding team will be led by the EPIS study PI, an experienced PhD-level mixed-methods researcher. A total of 3 coders, 2 research assistants with, at minimum, a baccalaureate degree, and 1 postdoctoral fellow with qualitative coding experience will code all the data. Coders will undergo initial training to familiarize themselves with the EPIS model, its constructs, and the operational definitions developed for the study. Coders will also be trained in the analytic approach, including the coding software. Coders will first collaboratively code 6 interviews (3 site PI and 3 key implementers) to familiarize themselves with the data and finalize the working codebook. An initial assessment of intercoder reliability will be conducted on 2 interviews (1 site PI and 1 key implementer). Coders will not be released for independent coding unless their intercoder reliability is at a minimum of 0.60 or higher as assessed by Cohen kappa [33]. To ensure intercoder reliability is maintained, a random selection of 30% of the interviews will be co-coded to ensure that the kappa coefficient remains 0.60 or higher [33]. After each intercoder reliability assessment, coders will meet to discuss and resolve coding discrepancies. Finally, the coding team is supported by 3 consultants with expertise in IS and/or HIV qualitative research.

The coded data will be comparatively analyzed both within and across time to examine differences at the setting and provider-level in quality and extent of EBP implementation. Examining the segments of text that are associated with differences in the frequency of categories between, for example, high-fidelity and low-fidelity sites, and examination of patterns in the presence and absence of thematic categories will allow us to provide empirically grounded explanations for differences in study outcomes.

#### Quantitative Analysis Plan

Analysis will begin by examining the psychometric properties, for example, internal consistency reliability using Cronbach alpha for all scales and subscales of established measures. Measures demonstrating insufficient reliability (eg, internal consistency <.70) in the study sample will be further examined with an exploratory factor analysis using Promax oblique rotation. Items with loadings <.40 or strong cross-loadings may be excluded for further analyses. Intercorrelations among items within each subscale and subscales within each measure will be examined; the correlation among measures will also be examined. Once reliability in the sample is established, descriptive analyses will be used to summarize the inner and outer contextual factors within and across sites and by informant (eg, site PIs and key implementers; clinical care providers and administrative staff). At baseline, we will examine mean differences in perceptions of intervention fit and attitudes toward EBP across site PIs and key implementers. We will also assess how perceptions vary as a function of Implementer demographics. At each follow-up, a comparison of changes in the inner and outer context factors over time (ie, from baseline to postintervention and sustainment) using a multivariate analysis will be conducted. Mixed linear effects models, adjusting for covariates, including age, time in position, role in clinic, experience level, and site-level factors, will be used to explore the impact these factors have on the overall implementation and sustainability of Scale It Up projects across sites and patient outcomes.

#### Mixed-Methods Analysis Plan

To offer findings in ways that move beyond the particularistic view of EBP implementation within the sites, once all of the data are coded across all time points, we will adopt the innovation profile approach [34] originally developed for classroom research. The approach results in a multidimensional rubric to classify where an organization is in the process of developing its capacity to engage in a particular set of activities, in this case, the integration of EBPs into routine patient care. The dimensions and subdimensions of the matrix we develop, as well as descriptions of the behavioral indicators of exemplary, intermediate, emerging, and low capacity to integrate EBPs, will be derived from aggregating the data produced during the analysis to the site level. These data will be integrated with quantitative fidelity data collected by the intervention protocol teams with equal weight given to qualitative and quantitative data sources [35]. We will follow best practices for conducting mixed-method designs in the health sciences as outlined by the Office of Behavioral and Social Sciences Research [36]. These include employing rigorous procedures in the methods of data collection and analysis and integrating the multiple sources of data toward the goal of obtaining rich, descriptive output.

## Results

**Figure 1:**
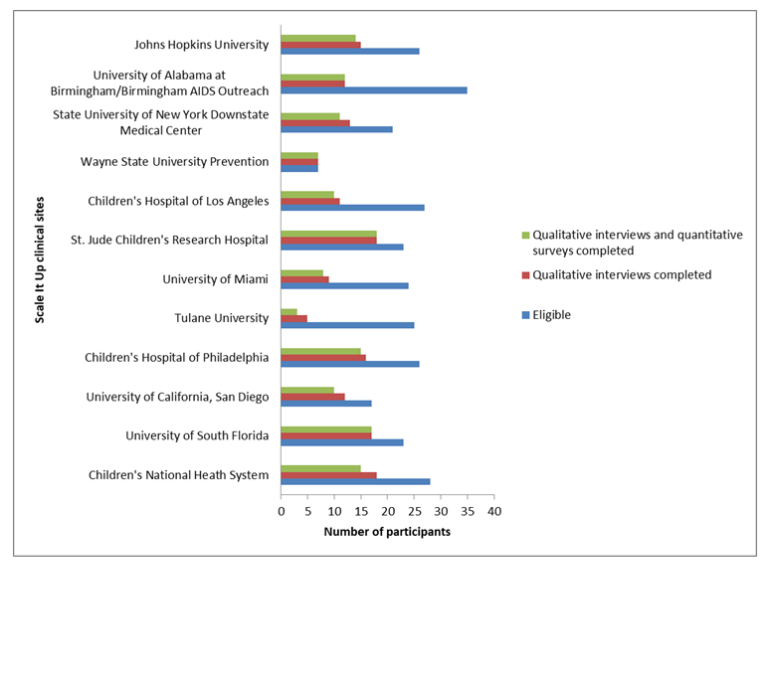
Total participant enrollment status per site till May 2018.

EPIS data collection was launched in June 2017 and, at the writing of this paper, the first phase (Preparation) of data collection has concluded, and analyses are underway. A total of 140 of 282 eligible stakeholders completed both components of the first EPIS data collection. The baseline data collection window closed with a small proportion of providers (13, 8.5%) having partially completed the baseline assessment, that is, the qualitative component was completed, and the quantitative survey remains outstanding. About 20% (56) declined to participate and the remaining stakeholders did not respond to the enrollment invitation before the closure of the baseline data collection window. Figure 1 illustrates completion rates by site. Follow-up data collections are scheduled to begin in March 2019 (Implementation) and March 2020 (Sustainment).

## Discussion

### Protocol Goal

Although EBPs have demonstrated success in the academic setting, many challenges can prevent an EBP’s successful implementation and sustainment in real-world clinical contexts. The goal of the EPIS IS study is to generate knowledge about the barriers and facilitators to the implementation and sustainment of EBPs into adolescent HIV prevention and clinical care settings. Understanding the factors that impact organizations, clinics, and practitioners throughout the EBP implementation process will facilitate the adoption of EBPs by tailoring implementation to fit within the needs and culture of the organization and/or clinic.

### Limitations

The EPIS sample is limited to the 13 participating ATN clinics and the medical providers and staff with direct patient contact within these clinical settings. These participants may not be representative of service providers in other contexts. This study and the Scale It Up program are focused on the implementation of EBPs in multidisciplinary adolescent HIV settings. The EPIS model was developed in child welfare [6,37] and has begun to be applied to other service sectors including behavioral health care [38] and juvenile justice [10]. In general, the findings from this research will add to the growing literature on IS and particularly the EPIS model, which will facilitate translation to other clinical settings.

### Implications

This study is the first IS study of EBP implementation in adolescent HIV settings. The knowledge gained from the EPIS study will strengthen the implementation and sustainment of EBPs in both adolescent prevention and clinical care contexts by offering insights into the barriers and facilitators of successful EBP implementation and sustainment in real-world clinical contexts.

## Abbreviations

ATN: Adolescent Medicine Trials Network for HIV/AIDS Interventions
DLS: Director Leadership Scale
EBP: evidence-based practice
EBPAS: Evidence-Based Practice Attitude Scale
EBPAS-50: Evidence-Based Practice Attitude Scale-50
EPIS: Exploration, Preparation, Implementation, and Sustainment Model
ICBS: Implementation Citizenship Behavior Scale
ICS: Implementation Climate Scale
ILS: Implementation Leadership Scale
IS: implementation science
MI: motivational interviewing
OCM: Organizational Climate Measure
PI: principal investigator
POS: Perceived Organizational Support Scale
